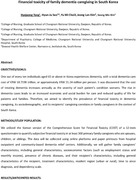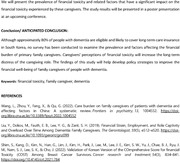# Financial toxicity of family dementia caregiving in South Korea

**DOI:** 10.1002/alz.084501

**Published:** 2025-01-09

**Authors:** Hyejeong Yang, Hyun‐Ju Seo, YuMi Choi, Jeong Lan Kim, Seong Min Kim

**Affiliations:** ^1^ Chungnam National University, Daejeon, Daejeon Korea, Republic of (South); ^2^ Chungnam National University Hospital, Daejeon, Daejeon Korea, Republic of (South); ^3^ Dowool Health Welfare Center, Namwon Korea, Republic of (South)

## Abstract

**Background:**

One out of every ten individuals aged 65 or above in Korea experiences dementia, with a total dementia care cost of KRW 18.7198 trillion, or approximately KRW 21.24 million per person. It was discovered that the cost of treating dementia increases annually as the severity of each patient’s condition worsens. The rise in dementia cases leads to an increased economic and social burden for care and reduced quality of life for patients and families. Therefore, we aimed to identify the prevalence of financial toxicity in dementia caregiving, its sociodemographic, and its recipients’ caregiving correlates in family caregivers in the context of South Korea.

**Method:**

We utilized the Korean version of the Comprehensive Score for Financial Toxicity (COST) of a 12‐item questionnaire to quantify subjective financial toxicity in at least 300 primary family caregivers who are spouses, children, or siblings. The data will be collected using online platforms and paper printouts from hospital outpatient and community‐based dementia relief centers. Additionally, we will gather family caregivers’ characteristics, including general characteristics, socioeconomic factors (such as employment status and monthly income), presence of chronic diseases, and their recipient’s characteristics, including general characteristics of the recipient, treatment characteristics, resident region (urban or rural), time to since diagnosis, and dependency scale.

**Result:**

We will present the prevalence of financial toxicity and related factors that have a significant impact on the financial toxicity experienced by these caregivers. The study results will be presented in a poster presentation at an upcoming conference.

**Conclusion:**

Although approximately 80% of people with dementia are eligible and likely to cover long‐term care insurance in South Korea, no survey has been conducted to examine the prevalence and factors affecting the financial burden of primary family caregivers. Caregivers' perceptions of financial toxicity will increase the long‐term distress of the caregiving role. The findings of this study will help develop policy strategies to improve the financial well‐being of family caregivers of people with dementia.